# Theory of Flexible Polymer Networks: Elasticity and Heterogeneities

**DOI:** 10.3390/polym12040767

**Published:** 2020-04-01

**Authors:** Sergey Panyukov

**Affiliations:** P. N. Lebedev Physics Institute, Russian Academy of Sciences, Moscow 117924, Russia; panyukov@lpi.ru

**Keywords:** elastomers, polymer networks, elastic modulus, loops, heterogeneities

## Abstract

A review of the main elasticity models of flexible polymer networks is presented. Classical models of phantom networks suggest that the networks have a tree-like structure. The conformations of their strands are described by the model of a combined chain, which consists of the network strand and two virtual chains attached to its ends. The distribution of lengths of virtual chains in real polydisperse networks is calculated using the results of the presented replica model of polymer networks. This model describes actual networks having strongly overlapping and interconnected loops of finite sizes. The conformations of their strands are characterized by the generalized combined chain model. The model of a sliding tube is represented, which describes the general anisotropic deformations of an entangled network in the melt. I propose a generalization of this model to describe the crossover between the entangled and phantom regimes of a swollen network. The obtained dependence of the Mooney-Rivlin parameters $C_{1}$ and $C_{2}$ on the polymer volume fraction is in agreement with experiments. The main results of the theory of heterogeneities in polymer networks are also discussed.

## 1. Introduction

Polymer networks and gels belong to a unique class of materials that have the properties of both solids and liquids. Such “soft solids” are elastically deformed at macroscopic scales, while at short distances and short times the network strands experience liquid fluctuations, which are responsible for the exceptional properties of polymer networks, such as the reversibility of huge deformations (when stretched up to 3000%, see [1]).

In this work, I review the main microscopic (molecular) approaches to the description of the elasticity of flexible polymer networks and establish relationships between these approaches. Phantom networks are considered in Section 2, and networks with topological entanglements are studied in Section 3. Spatial heterogeneities developed in swollen and deformed polymer networks are investigated in Section 4.

The classical theory proposed by Flory more than half a century ago, Ref. [2] suggests that polymer networks have a tree-like structure. Although this theory takes into account the presence of loops in the network, it is assumed that they all are infinite in size. Such an approach is insufficient to describe the thermodynamics of polymer networks with loops of finite size, and explicit consideration of these loops is required. The mathematical model of such networks is based on the replica method. This approach takes into consideration that the properties of the network depend not only on the “current” conditions in which the network is placed but also on the conditions of its preparation since it is these conditions that determine the molecular structure of the formed network. The replica approach takes into account the excluded volume interaction of linear chains both at preparation and experiment conditions by generalizing the theory of the “n→0-component” field $\varphi^{4}$ proposed by de Gennes. The main properties of the order parameter φ introduced for the description of such “soft solids” are discussed in Section 2.

The conformations of the chains in the network are described by the combined chain model [3]. This model is based on the concept of virtual chains, which determine the effective elasticity of the tree-like structures in the network. In polydisperse networks, the distribution of virtual chains is also polydisperse. I show that the order parameter in the replica network model stores information about this distribution. The found solution of the replica model is used to calculate the distribution function of the lengths of virtual chains in polydisperse networks.

The structure of real networks differs significantly from the classical picture of ideal trees. Typical loops of such networks have finite dimensions and strongly overlap with each other. The impact of such loops on the conformations of the network chain is described by the extended combined chain model [4]. I discuss the connection of this model with the predictions of the replica theory, which offers an analytical description of such mutually overlapping loops. Along with typical loops, the network may contain topological defects, which are also taken into account by the replica method. The condition for the loss of elasticity by polymer networks, both due to the presence of topological defects and near the gel point is discussed.

With an increase in the length of polymer chains, the effects of topological entanglements become significant. The properties of entangled polymer liquids are significantly different from the properties of entangled soft solids—polymer networks. While non-concatenated rings in the melt are compacted into fractal loopy globules [5], internal stresses in stretched polymer networks prevent such a collapse. Entangled networks are described by the non-affine tube model that generalizes the concept of virtual chains to the case of entangled polymer systems [6]. I discuss the physics of deformation of network strands in this model in Section 3.

Anisotropic deformations of the entangled network lead to slippage of chains along the contour of the entangled tube, which is described by the slip tube model [3]. This model suggests the additivity of the phantom and entangled contributions to the free energy of the network. Although this additivity approximation describes well the uniaxial deformations of the network in the melt, it cannot be used to describe the crossover of the Mooney Rivlin dependence between entangled and phantom deformation regimes observed during swelling of the polymer network. I propose the generalization of the slip tube model to account for this crossover and show that the obtained concentration dependences of the Mooney-Rivlin parameters $C_{1}$ and $C_{2}$ are in agreement with the experimental data.

The irregularity of the molecular structure of polymer networks leads to the presence of spatial inhomogeneities. The main results of the theory of heterogeneities in polymer networks are discussed in Section 4.

## 2. Phantom Networks

The polymer network is obtained by crosslinking linear chains. In models of phantom polymer networks, it is assumed that in the process of thermodynamic fluctuations, polymer chains can freely “pass” through each other. The phantom approximation works well only for not too long network strands; otherwise, topological restrictions related to the mutual impermeability of polymer chains should be taken into account. In this section, I discuss the main approaches to the theoretical description of phantom networks. The elastic free energy of a network of Gaussian chains is quadratic in the deformation ratios $\lambda_{\alpha }=L_{\alpha }/L_{0\alpha }$ of the network dimensions along the principal axes of deformation α=x,y,z in the deformed state ($L_{\alpha }$) and under conditions of its preparation ($L_{0\alpha }$):

(1) $$F_{ph}=G\sum_{\alpha }\frac{\lambda_{\alpha }^{2}-1}{2}$$

The interaction of strand monomers is usually taken into account by imposing the incompressibility condition

(2) $$\lambda_{x}\lambda_{y}\lambda_{z}=1/\phi .$$

where ϕ is the polymer volume fraction. In the case of uniaxial deformation we have

(3) $$\lambda_{x}=\phi^{-1/3}\lambda ,\;\lambda_{y}=\lambda_{z}=\phi^{-1/3}\lambda^{-1/2}.$$

In the affine network model, it is assumed that the ends of each network strand are pinned to a “non-fluctuating elastic background” which deforms affinely with the network surface [3]. The elastic modulus of such a network is proportional to the density ν of its strands,

(4) G=kTν

and kT is the thermal energy. Equation (4) is attractive for its simplicity. However, in real networks, the strand ends are not fixed in space—they are connected to neighboring strands through cross-links. The elastic modulus *G* depends on the molecular structure of the network and the polydispersity of its strands. At first glance, the “bookkeeping” of the order of connection of all network strands with each other and the numbers of monomers of each of these strands seems impossible for networks of macroscopic sizes. However, this is precisely what the replica method does.

### 2.1. Replica Method

The replica method was proposed a long time ago [7] and was used later to calculate correlation functions of density fluctuations in polymer networks [8]. The main goal of this approach is to build a mathematical model that takes into account most of effects inherent to real polymer networks. Unlike widespread numerical simulations, which can also take these effects into account, the replica method is an analytical theory, which is more amenable for understanding network elasticity as a function of many different molecular features.

**Figure 1:**
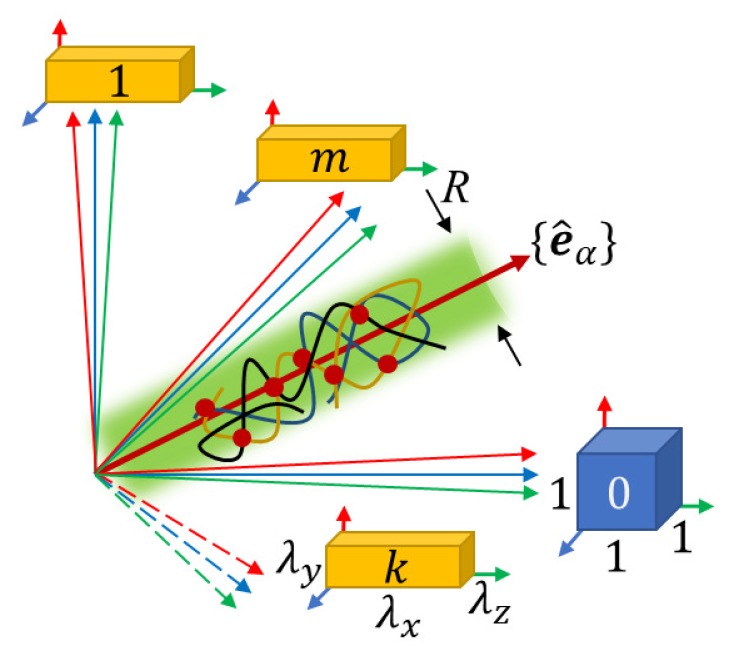
The replica system consists of the initial system (k=0)—the polymer network at preparation conditions (blue cube) and *m* identical replicas of the final system (k=1,…,m)—the network, deformed by factors $\lambda_{\alpha }$ along the principal axes α=x,y,z of deformation (yellow cuboids). The polymer network in the replica space of dimension 3(1+m) is mainly localized inside the (green) cylinder with the diameter $R≃a{\bar{N}}^{1/2}$ directed along unit vectors $\left\{{\hat{e}}_{\alpha }\right\}$, corresponding to affine deformation of the polymer. The networks (and all their monomers and strands) in the initial system and any of the *m* replicas of the final system are the projections of the network in the replica space onto the corresponding subspaces k=0,…,m.

When the polymer network is deformed, its structure remains unchanged, formed under the conditions of its preparation. The main idea of the replica approach is to consider the polymer network in an expanded space with coordinates $\hat{x}=\left(x^{\left(0\right)},x^{\left(1\right)},\ldots ,x^{\left(m\right)}\right)$ of dimension 3(1+m). The replica space consists of an “initial system” under conditions of the network preparation (with coordinates $x^{\left(0\right)}$) and *m* replicas of the “final system” under experimental conditions (with coordinates $x^{\left(k\right)}$, k=1,…,m), see Figure 1.

In each of these systems, the polymer network with all of its monomers and bonds is the projection of the network in the replica space onto the corresponding subspaces. Therefore, in all these systems, the network structure is exactly the same, while the conformations of network strands can vary significantly. The free energy *F* of the final system (a deformed network) is expressed through the analytic continuation to m=0 of the free energy $F_{m}$ of the replica system [8,9]:

(5) $$F=\lim_{m\to 0}\frac{dF_{m}}{dm}.$$

### 2.2. Liquid-Solid Order Parameter

According to de Gennes, polymer networks are “soft solids”, which emphasizes the presence of liquid and solid-state degrees of freedom interacting with each other. In the Landau approach, solids are described by an order parameter—a number (in general case, a tensor), equal to zero for a liquid and nonzero only in the solid phase. Unlike ordinary low molecular weight solids, the properties of polymer networks depend on the characteristics of both the initial and final systems. Therefore, the order parameter for polymer networks is not a number, but a function of the difference of coordinates $x_{\alpha }^{\left(k\right)}-\lambda_{\alpha }x_{\alpha }^{\left(0\right)}$ of final and initial systems. This dependence reflects the dual nature of polymer networks: At scales larger than the characteristic size of the network cycles, the network deforms affinely as an ordinary solid, see Figure 1. On a smaller spatial scale, the network chains fluctuate in space and have conformations similar to the chains in a polymer liquid. The unique properties of polymer networks are determined by the interaction of their solid-state and liquid degrees of freedom [10].

In the liquid phase, the order parameter is independent of coordinates. In the case of Gaussian polymer networks, the order parameter $\varphi \left(\varsigma \right)$ is a function of a single variable

(6) $$\varsigma =\frac{1}{2}\left[{\hat{x}}^{2}-\sum_{\alpha }{\left({\hat{e}}_{\alpha }\hat{x}\right)}^{2}\right],$$

where ${\hat{e}}_{\alpha }$ is a unit vector along the direction $x_{\alpha }^{\left(k\right)}=\lambda_{\alpha }x_{\alpha }^{\left(0\right)}$ of the affine deformation in the replica space. At ς=0 (that is, at $x_{\alpha }^{\left(k\right)}=\lambda_{\alpha }x_{\alpha }^{\left(0\right)}$), the order parameter $\varphi \left(0\right)$ is determined only by the conditions of the network preparation and does not depend on the experimental conditions. This last dependence is encrypted in the dimensionless order parameter $\chi \left(t\right)=\varphi \left(\varsigma \right)/\varphi \left(0\right)$ which is the function of the dimensionless variable t=ς/R. In the case of well-developed networks obtained by crosslinking polymer chains with *f*-functional monomers far beyond the gel point, the characteristic radius is $R≃b{\bar{N}}^{1/2}$, see Figure 1. Here *b* is the monomer size and $\bar{N}$ is the average number of network strand monomers.

In the case of monodisperse networks, the function $\chi \left(t\right)$ was calculated by Edwards (see Equation (3.19) of Ref. [11]):

(7) $$\chi \left(t\right)=e^{-\frac{f-2}{f-1}t}$$

In the case of polydisperse networks, this function $\chi \left(t\right)$ is determined by the differential equation (see Appendix A)

(8) $$t\chi^{\prime \prime }\left(t\right)=\chi \left(t\right)-\chi^{f-1}\left(t\right)$$

For large t≫1, the function $\chi \left(t\right)$ decreases more slowly compared to the monodisperse case (7), as a stretched exponent

(9) $$\chi \left(t\right)∼t^{1/4}e^{-2t^{1/2}}$$

For small *t*, the solution of Equation (8) can be approximated by a power function

(10) $$\chi \left(t\right)≃{\left(1+ct\right)}^{-k}$$

Substituting it in Equation (8) and equating to zero the first two expansion coefficients (*t* and $t^{2}$) in powers of *t*, we find

(11) $$k=\frac{3}{f-2},\;c=\frac{{\left(f-2\right)}^{2}}{f+1}$$

The replica method is also called “replica trick” because of the hidden meaning of its mathematical constructions. As will be shown below, Equation (8) describes the relationship between distribution functions of the molecular trees that characterize the structure of the polymer network. Note that Equation (8) is obtained by estimating the Hamiltonian (A2) in the Appendix A using the steepest descent method. Therefore, it corresponds to the mean-field approximation, which works due to the presence of a large number of overlapping network strands.

### 2.3. Overlap Parameter

Like in liquid polymer systems, in a typical network there are many overlapping network strands. An important characteristic of the network is the overlap parameter, which is defined as the number of network strands within the volume $R^{3}$ pervaded by one network strand. In the case of a solution of chains with monomer density $\rho^{\left(0\right)}$, $P^{\left(0\right)}≃\rho^{\left(0\right)}R^{3}/N$. In Gaussian networks, the size of the network strand with Kuhn length *b* is $R≃bN^{1/2}$, and

(12) $$P^{\left(0\right)}≃\rho^{\left(0\right)}b^{3}N^{1/2}$$

with a large parameter $P^{\left(0\right)}$, polymer networks obtained far from the gel point can be considered as $P^{\left(0\right)}$ overlapping yet independent “elementary” networks, the strands of which consist of *N* monomers. There is on the order of one network strand per volume $R^{3}$ in the “elementary” network, and its elastic modulus is estimated as $G_{elem}≃kT/R^{3}$, kT is the thermal energy. Since the real network consists of $P^{\left(0\right)}$ overlapping elementary networks, its elastic modulus is $P^{\left(0\right)}$-times larger [12]:

(13) $$G=P^{\left(0\right)}G_{elem}≃kT\rho^{\left(0\right)}/N$$

The polymer network can also be represented as $P^{\left(0\right)}$ polymer trees (or “layers”) overlapping with each other. The perfect network model assumes an infinite number of layers, and also that these trees have an infinite number of generations. In real networks $P^{\left(0\right)}<P_{e}$ is finite and not too large (the entanglement overlap parameter $P_{e}∼$ 20–30, see Section 3 below), and the different root trees are interconnected by loops. The number of network strands forming a closed loop of minimum length, which binds together different layers of the network, depends logarithmically on the overlap parameter [4],

(14) $$l\approx \frac{1}{f-1}\ln P^{\left(0\right)}$$

The case $P^{\left(0\right)}\approx 1$ with the loop size l≈1 describes the “elementary networks” in Equation (13). In accordance with the results of numerical simulations [13], the loop length *l* decreases with dilution and with increasing functionality *f* of cross-links. Note that the minimal size loops do not directly determine the network elasticity since each elastically effective chain is simultaneously part of a large number of loops. The cumulative effect of these typical loops of the network is self-averaged, and can be described in the effective mean-field approximation, see Equation (37) below.

The overlap parameter also determines the density fluctuation of the network obtained in the case of incomplete conversion above the gel point, at $p>p_{c}$. In the mean-field approximation, the density of gel monomers is

(15) $$\rho_{g}^{\left(0\right)}≃\left(p-p_{c}\right)\rho^{\left(0\right)},$$

and its connectivity radius is estimated as $\xi ≃b{\left[N/\left(p-p_{c}\right)\right]}^{1/2}$. Fluctuations in the number of monomers of the polymer network on the scale of the connectivity radius occur by attaching or detaching sol molecules with a characteristic number of monomers

(16) $$L≃N/{\left(p-p_{c}\right)}^{2}$$

Therefore, relative fluctuations in the gel density are estimated as

(17) $$\frac{\delta \rho_{g}^{\left(0\right)}}{\rho_{g}^{\left(0\right)}}≃\frac{L}{R_{bb}^{3}\rho_{g}^{\left(0\right)}}≃\frac{1}{{\left(p-p_{c}\right)}^{3/2}P^{\left(0\right)}},$$

In the case of a large overlap parameter, these fluctuations are small outside the narrow Ginzburg region,

(18) $$p-p_{c}\gg {\left(P^{\left(0\right)}\right)}^{-2/3}$$

### 2.4. Combined Chain Model: Monodisperse Networks

**Figure 2:**
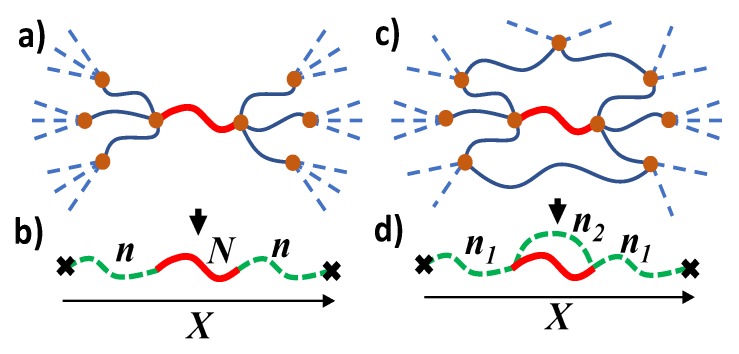
(**a**) The model of perfect network with tree-like connection of network strands (solid curves) and infinite size loops (dotted lines). (**b**) A network strand with *N* monomers can be considered as a part of the combined chain, connected through two effective chains with *n* monomers (dashed green curves) to the non-fluctuating background. (**c**) Actual network with finite size loops. (**d**) The combined chain consists of three effective chains. The filled circles and crosses indicate cross-links and nonfluctuating background, respectively. The end-to-end vector of the combined chain X deforms affinely with network deformation.

The elasticity of the polymer network has an entropic origin and is determined by the change in strand conformations during the network deformation. The conformations of a strand depend on its interaction with the elastic environment, which can be described by virtual chains attached to the ends of this strand. The network strand together with the two virtual chains is called a combined chain. The ends of this combined chain are attached to an affine deformable non-fluctuating background, see Figure 2b.

In the perfect network model, it is assumed that the network has a tree structure, whereas all its cycles are of infinite size, see Figure 2a. Each of the trees attached to the ends of the network strand is modeled by a virtual chain. The effective number *n* of its monomers is related to the numbers $n_{i}$ of virtual chain monomers on the next generation of the tree i=1,…,f−1 and the corresponding numbers of strand monomers $N_{i}$ as

(19) $$\frac{1}{n}=\sum_{i=1}^{f-1}\frac{1}{m_{i}},\;m_{i}=N_{i}+n_{i}$$

In the case of a monodisperse network with a fixed number $N_{i}=N$ of monomers of its strands, all $n_{i}=n$ and the solution of Equation (19) determines the number of monomers of the virtual chain [14]

(20) $$n=N/\left(f-2\right)$$

The average vector R between the ends of the network strand is determined from the force balance condition. In the case of a monodisperse network,

(21) $$R=\frac{X}{1+2n/N}$$

where *n* is the number of monomers of the virtual chain, Equation (20). The mean square fluctuation of the vector R is determined by the parallel connection of the real chain and two serially-connected virtual chains

(22) $$\langle {\left(\Delta R\right)}^{2}\rangle =\frac{b^{2}}{1/N+1/\left(2n\right)}=\frac{2}{f}b^{2}N$$

The vector X between the ends of the combined chain is deformed affinely with macroscopic network deformation. The elastic modulus of the perfect network with monomer density $\rho^{\left(0\right)}$ and chain concentration $\rho^{\left(0\right)}/N$ is

(23) $$G=\frac{kT\rho^{\left(0\right)}/N}{1+2n_{1}/N}=kT\nu \left(1-\frac{2}{f}\right)$$

Here $\nu =\rho^{\left(0\right)}/N$ is the concentration of strands and ${\left(1+2n_{1}/N\right)}^{-1}$ is the strand fraction of the combined chain. A comparison of expressions (23) and (4) shows that the elastic modulus of the perfect network is less than the modulus of the affine network by a factor of 1−2/f. It is generally accepted that the 2/f factor is associated with the presence of fluctuations in the strand size, which are also proportional to this factor (see Equation (22)). In fact, fluctuations have nothing to do with it [15]: an exact elastic modulus of Gaussian phantom network is different from that of the perfect network model and coincides with the elastic modulus of the classical non-fluctuating grid, in which each strand is replaced by a corresponding elastic thread with the same elastic stiffness coefficient [16]. This coincidence is due to affine deformation of the average distances between the cross-links in such networks.

Equation (23) can be recast in more general form with the network modulus proportional to the difference of the number densities of network strands ν and cross-links μ=2ν/f, since there are f/2 strands per crosslink [14]:

(24) $$G=kT\left(\mu -\nu \right)$$

This expression is quite universal; with general densities ν and μ, it describes polydisperse networks, as well as networks with incomplete conversion, prepared above the gel point far from the Ginzburg region defined by Equation (18). In general, μ−ν makes a sense of the cyclic rank of the network of unit volume. To define it, mentally cut one of the strands so that the network does not break into two disconnected parts. The cyclic rank is equal to the maximum number of such cuts, and it makes sense the number of independent network loops. An important limitation of expression (24) is the assumption of a model of perfect networks about the infinite sizes of all of the network cycles.

In real networks, not all loops transmit stress in the network. For example, primary loops, connected to the network at only one crosslink, are not capable of permanently supporting a stress. To exclude the contribution of such elastically ineffective loops and dangling chain ends, it was proposed to account in Equation (24) only for the elastically effective strands and crosslinks. Elastically effective strands deform and store elastic energy upon network deformation [17]. Elastically effective crosslinks connect at least two elastically effective strands [18]. The elastic modulus of real networks can significantly differ from such a modified expression (24), since each of finite size loops is characterized by its “elastic effectiveness”, which depends on the type of the loop. The replica method provides a universal tool for accounting for such effects, see Equation (37) below.

### 2.5. Combined Chain Model: Polydisperse Networks

Real networks are polydisperse, and therefore, the numbers of monomers *n* of virtual chains are random variables. Information on the distribution of the lengths of virtual chains is important for studying the distribution of tension in real chains of the network. Below I calculate the distribution function $p\left(n\right)$ of virtual chain lengths. The distribution of inverse variables s=1/n is described by the function

(25) $$q\left(s\right)=\frac{1}{s^{2}}p\left(\frac{1}{s}\right),\;s=\frac{1}{n}$$

Appendix B provides an algorithm for calculating this distribution for an arbitrary distribution $P\left(N\right)$ of the number of monomers *N* of the real network strands. The problem is reduced to solving a system of nonlinear integral equations. In the most interesting case of the exponential distribution $P\left(N\right)=e^{-N/\bar{N}}/\bar{N}$, the asymptotic behavior $n\gg \bar{N}$ of the solution of these equations can be found

(26) $$p\left(n\right)∼e^{-{\left(f-1\right)}^{2}n/\bar{N}}$$

Of course, knowledge of only asymptotic is not enough to describe conformations of a chain in the polydisperse network. The replica method offers a much more constructive approach, allowing us to calculate this function over the entire range of *n* values. One can show, generalyzing calculations of Ref. [9] that the distribution function $q\left(s\right)$ in Equation (25) is determined by inverse Laplace transform of the function $\chi^{f-1}\left(t\right)$ with the dimensionless order parameter $\chi \left(t\right)=\varphi \left(\varsigma \right)/\varphi \left(0\right)$,

(27) $$\int q\left(s\right)e^{-s\bar{N}t}ds=\chi^{f-1}\left(t\right)$$

Due to the boundary condition $\chi \left(0\right)=1$ for the function $\chi \left(t\right)$, the distribution function $q\left(s\right)$ is normalized to unity. To better understand the meaning of Equation (27), we substitute the exponential function $\chi \left(t\right)$ for the monodisperse networks, Equation (7), into this equation. The result is a δ-functional distribution,

(28) $$q\left(s\right)=\delta \left[s-\left(f-2\right)/N\right],$$

in accordance with expression (20) for the number of monomers n=1/s of virtual chains.

In the case of polydisperse networks, this correspondence, Equation (27), establishes a connection between the standard method of distribution functions and the replica approach. Since $\chi^{f-1}\left(t\right)$ is the Laplace transform of the convolution function ${\tilde{\chi }}^{\left(f-1\right)}\left(t\right)$ (see Appendix B), the function $\chi \left(t\right)$ is the Laplace transform of the distribution function $\tilde{\chi }\left(s^{\prime }\right)$ defined by Equation (A7). Equation (8) for this function corresponds to Equation (A8) of the replica approach for the exponential distribution of the numbers of monomers of real strands.

Substituting the asymptotic expression (9) for the function $\chi \left(t\right)$, into Equation (27), we find a more accurate asymptotic expression for the distribution function

(29) $$p\left(n\right)≃\frac{1}{\bar{N}}{\left(\frac{n}{\bar{N}}\right)}^{f/2-1}e^{-{\left(f-1\right)}^{2}n/\bar{N}},\;n\gg \frac{\bar{N}}{{\left(f-2\right)}^{2}}$$

Using the function $\chi \left(t\right)$ from Equation (10), we get

(30) $$p\left(n\right)≃\frac{c}{\bar{N}\Gamma \left[\left(f-1\right)k\right]}{\left(\frac{\bar{N}}{cn}\right)}^{k\left(f-1\right)+1}e^{-\frac{\bar{N}}{cn}}$$

Thus, the distribution $p\left(n\right)$ of virtual chains vanishes with all its derivatives as n→0 and decreases exponentially on the scale $\bar{N}/{\left(f-2\right)}^{2}$ for large *n*. Calculating the average number of monomers of the virtual chain with the distribution (30), we find

(31) $$\bar{n}≃\frac{f+1}{2f-1}\frac{\bar{N}}{f-2}$$

Note that the obtained distribution of the lengths of the virtual chains is narrower than the initial distribution of the lengths of the network strands.

### 2.6. Generalized Combined Chain Model

A perfect network assumes a tree-like structure on all spatial scales. In real networks, due to the excluded volume effect, trees cannot “grow” to infinity (the Malthus effect), since too large trees cannot fit in real 3D space. Therefore, in real networks, the size of a typical tree is finite, and the network consists of a large number of loops of finite length, see Figure 2c. They are strongly overlapped and interconnected with each other. The impact of such typical loops, which are responsible for the solid-state elasticity of the network, can be described by the generalized combined chain model.

In this model, in addition to two virtual chains of $n_{1}$ monomers, the combined chain includes an additional virtual chain of $n_{2}$ monomers, see Figure 2d. This virtual chain represents an effective elastic of all loops of finite size in the network, shunting the real strand of *N* monomers. The average end-to-end vector *R* of the network strand is related to the end-to-end vector *X* of the combined chain via the force balance condition, generalizing Equation (21):

(32) $$R=\frac{X}{1+2n_{1}\left(1/N+1/n_{2}\right)}.$$

In this model, the mean square fluctuation of the vector R is

(33) $$\langle {\left(\Delta R\right)}^{2}\rangle =\frac{b^{2}}{1/N+1/\left(2n_{1}\right)+1/n_{2}}=\frac{b^{2}}{1/N+1/\left(2n\right)}$$

The second equality can be treated as the contribution of two effective virtual chains connecting the network strand to the non-fluctuating background. They have renormalized number of monomers

(34) $$2n=\frac{2n_{1}n_{2}}{2n_{1}+n_{2}},$$

corresponding to parallel connection of two effective chains: with $2n_{1}$ and $n_{2}$ monomers.

The elastic modulus of this model is

(35) $$G=\frac{kT\rho^{\left(0\right)}/N}{1+2n\left(1/N+1/n_{2}\right)}\frac{1}{1+N/n_{2}}$$

The factor ${\left(1+N/n_{2}\right)}^{-1}$ is the fraction of energy related to the network strand when it is connected in parallel with the effective chain of $n_{2}$ monomers, see Figure 2d. This result can be rewritten in the form

(36) $$G=\frac{kT\rho^{\left(0\right)}}{N+2n}-\frac{kT\rho^{\left(0\right)}}{N+n_{2}}.$$

where 2n is the number of monomers of the effective virtual chains, Equation (34). The first term in this expression has the same meaning as for the perfect network in Equation (23). The negative second contribution in Equation (36) is due to the finite size of typical loops in the network. The number of monomers of the shunt virtual chain, $n_{2}$, can be calculated using the replica method.

### 2.7. Finite-Size Loops of Real Networks

In real networks, there are always cyclic defects of finite dimensions. Sparse structural defects can be taken into account within the framework of the perfect network model, assuming that they are connected with the affinely deformed non-fluctuating background through the root trees. These trees can be modeled by the virtual chains [3]. Since part of the network strands is “spent” on creating the cyclic fragment itself, the length of the effective chain linking this fragment to the elastic non-fluctuating background is different from Equation (20). In polydisperse networks, the distribution function of such virtual chains is determined by Equation (27), in which f−1 has the meaning of the number of branches on the first generation of this tree. In the ideal defect gas approximation, the contribution of the structural defects to the elastic modulus of a perfect network was calculated in works [19,20,21,22]. Note that typical network loops are ignored in this approximation, which takes into account only explicitly treated loops of small concentration.

In real networks, there are both loops, binding different network layers (see Equation (14)) and topological defects, which are not sparsely distributed. The replica method allows calculating the effect of both typical loops and cyclic defects of arbitrary concentration on the elasticity of the network. The elastic modulus of the network obtained by random end-linking polydisperse chains by cross-links with functionality *f* and concentration $\rho_{f}^{\left(0\right)}$ and cyclic fragments with functionality $\left\{f_{i}\right\}$ and arbitrary concentrations $\left\{x_{i}\rho_{f}^{\left(0\right)}\right\}$ is [4]

(37) $$G=kT\rho_{f}^{\left(0\right)}\frac{f/2-1+\sum_{i}\left(f_{i}/2-1\right)x_{i}}{1+\sum_{i}ix_{i}}-kTv^{\left(0\right)}\rho^{\left(0\right)}\frac{Q\left(0\right)}{2}$$

The functionality $f_{i}$ of the cyclic defect defined as the number of “external” network strands joined to the cyclic fragment. For the ring fragment with *i* “internal” strands and cross-links, $f_{i}\;=\;i\left(f-2\right)$, since two of the functional groups of each cross-link are involved in creating the ring. In the case of incomplete conversion, p<1 (but still far from the gelation threshold, |1−p|≪1), in expression (37), instead of functionalities *f* and $f_{i}$, their average values should be substituted, pf and $pf_{i}$. Such a dependence of the network elastic modulus on the conversion is in agreement with numerical simulations of Gaussian networks, performed in Ref. [16].

The fractions $x_{i}$ of cyclic defects (small fragments of the network with i⩾1 “internal” cross-links) depend on the type of reactions leading to the formation of the network [23,24]. All $x_{i}$ universally depend on one dimensionless parameter $x_{1}$ characterizing the conditions for network preparation [25]. The primary loops with concentration $x_{1}$ are tied to the network at only one crosslink. The denominator of the first term in expression (37) describes an increase in the effective number of monomers between cross-links due to primary loops, $\bar{N}\to \bar{N}\left(1+x_{1}\right)$. Therefore, although primary loops cannot bear shear stress in the network, they renormalize the length of the elastically effective chains, which deform and store elastic energy upon network deformation [7,9].

For general f>2, the first term in Equation (37) can be interpreted as the contribution of elastically effective network strands with renormalized number of monomers due to the presence of topological defects in the network—primary loops and cyclic fragments of finite size. Only this contribution is predicted in the classical model of phantom networks. Its expansion in a series in the parameters $x_{i}$ with accuracy up to first order terms reproduces the result of the so-called “network theory with strand pre-strain” [21]. At network preparation conditions, the strands of a cyclic fragment are contracted and the surrounding strands are stretched (pre-strained) with respect to Gaussian sizes of these chains [22]. These effects are “automatically” taken into account in the framework of the replica approach in Equation (37).

The last term in Equation (37) is always negative and gives an impact of a large number of typical loops of the network. The factor $Q\left(0\right)$ in the second term of expression (37) determines the probability of the formation of typical closed loops. These loops shunt elastically effective chains of the network, an effect that is taken into account by an additional effective chain in the model of the generalized combined chain in Figure 2d. Unlike perfect networks, the monomers of real networks mutually repel each other, “crowding out” the network loops on small length scales, see Figure 2. Therefore, the elasticity of a polymer network, Equation (37), explicitly depends on excluded volume parameter $v^{\left(0\right)}$, characterizing monomer interaction at the preparation conditions. This effect is ignored by the classical theory of polymer networks.

Note that the elastic modulus of the polymer network *G* in Equation (37) vanishes at a finite fraction $x_{1}$ of cyclic fragments. Such a network with overlap parameter $P^{\left(0\right)}\gg 1$ has tree-like connections of overlapped cyclic fragments with a finite monomer density. To better understand this result, we compare it with the more familiar case of a disordered low-molecular-weight solid. In this case, the network fragments cannot overlap since the overlap parameter $P^{\left(0\right)}=1$. Therefore, the elastic modulus turns to zero only at the point of percolation transition, at which the network density also vanishes.

A similar effect was observed in numerical simulations of networks obtained near the gel point, which is an analog of the percolation transition in low-molecular-weight solids. It is shown that the elastic modulus of such networks vanishes at a finite density of the network monomers [26]. One can estimate the corresponding shift in critical conversion $p_{c\mu }$ at which G=0, compared with the gel point at the conversion $p_{c\gamma }$, at which the average degree of polymerization of soluble molecules diverges. For end-linked networks, the looping probability is $Q\left(0\right)≃{\left(bN^{1/2}\right)}^{-3}∼1/\left(NP^{\left(0\right)}\right)$. Dropping all the numerical factors, we find from Equation (37) the mean-field estimate for the elastic modulus

(38) $$G≃kT\frac{\rho_{g}^{\left(0\right)}}{L}-kT\frac{\rho_{g}^{\left(0\right)}}{NP^{\left(0\right)}}$$

Here $L≃N/{\left(p-p_{c\gamma }\right)}^{2}$ and $\rho_{g}^{\left(0\right)}≃\left(p-p_{c\gamma }\right)\rho^{\left(0\right)}$ is the density of gel monomers, see Equations (16) and (15). Therefore, G=0 at $p_{c\mu }-p_{c\gamma }∼1/\sqrt{P^{\left(0\right)}}$, in accordance with the result of numerical simulations [26]. A more rigorous description of this effect requires the inclusion of fluctuation corrections. Inside the strongly fluctuating Ginzburg region, Equation (18), the overlap parameter of elastically effective chains is small (which is why strong density fluctuations develop in this region), and they can be described by critical exponents of percolation theory, see Reference [26].

## 3. Entangled Networks

The physics of topological entanglements is perhaps one of the most “entangled” sections of physics of polymer networks since it is impossible to give a sufficiently rigorous and constructive formalism that adequately describes entanglements for networks of macroscopic size. Therefore, the consideration of topological entanglements is usually carried out using some uncontrolled assumptions, allowing to switch from substantially “multi-chain” problem to considering the conformations of one network strand. The transfer of elastic shear stresses from individual strands of a network to its solid-state degrees of freedom (represented by the affinely deformed non-fluctuating background) is described by virtual chains. In phantom networks, such a transfer is performed only through cross-links at the strand ends. In entangled networks, the stress is carried by all entangled segments of the strand due to its topological interaction with the loops formed by adjacent network strands.

Entangled segments are characterized by the large overlap parameter

(39) $$P_{e}≃\rho^{\left(0\right)}b^{3}N_{e}^{1/2}$$

where $N_{e}$ is the number of monomers of the entangled strand. In the case of flexible polymers, the parameter $P_{e}≃$ 20–30. The number of monomers in an entanglement strand $N_{e}$ and the overlap parameter $P_{e}$ depend on the polymer chemistry [27].

As shown in [5], in the melt of non-concatenated rings the condition $P_{e}=const$ is satisfied at all scales down to the distance $a_{0}≃bN_{e}^{1/2}$ between adjacent entanglements. Entangled loops of different sizes overlap with similar size loops at the same overlap parameter $P_{e}$. As a result, ring molecules are packaged into the self-similar structures called fractal loopy globules. Unlike ordinary globules, in which chains with Gaussian statistics experience random reflections from the surface of the globule, the ring chains in the fractal loopy globules are not Gaussian and form loops at all scales, starting from a size

(40) $$a_{0}≃bN_{e}^{1/2}$$

of the entangled segment. Ring sections with the number of monomers $g<N_{e}$ smaller than entanglement strands are still Gaussian with size $r≃bg^{1/2}$. Larger subsections of a ring are compressed with fractal dimension $d_{f}$=3 and size

(41) $$r≃a_{0}{\left(g/N_{e}\right)}^{1/3},\;a_{0}<r<R$$

up to the size *R* of a loopy globule in a melt

(42) $$R≃bN^{1/3}N_{e}^{1/6}$$

**Figure 3:**
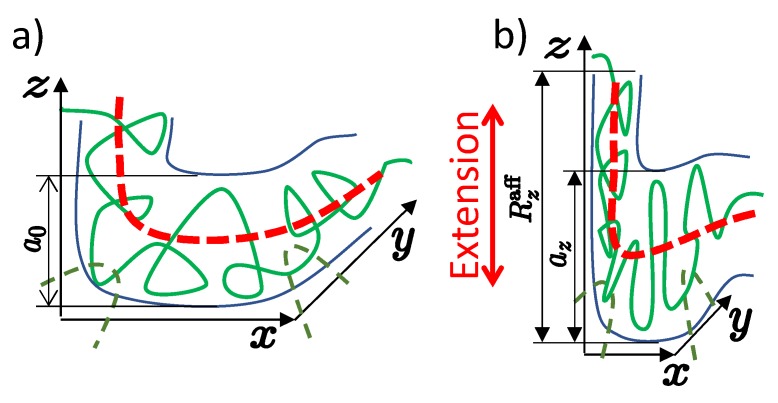
Nonaffine tube model. (**a**) In an undeformed state, the chain fluctuates in entangled tube of diameter $a_{0}$ (**b**) The tube diameter $a_{z}$ in the stretching direction is less than the affine length $R_{z}^{aff}$. The dashed lines show the trajectory of the primitive path, which is obtained by smoothing the affine deformed coordinates of the original Gaussian chain on the tube diameter scale *a*.

Large typical values of the parameter $N_{e}\gg 1$ allow developing the mean-field theory of entanglements in polymer networks, since the total effect of large number $P_{e}\gg 1$ of loops interacting with the entanglement segment is effectively averaged. The strands of the entangled polymer networks are confined in an effective entangled tube with a diameter *a*, which, under the conditions of the network preparation, is equal to the size $a_{0}$ of the entangled segment. The tube rotates by a random angle of about $90^{\circ }$ on the scale $a_{0}$, see Figure 3a.

In the case of networks obtained by crosslinking a melt of linear chains, the presence of a large number of overlapping entangled chains allows using the mean-field approximation. In this approximation, topological interactions are described by virtual chains attached to all monomers (s=1,…,N) of the chain with coordinates x(s). The elastic energy of Gaussian virtual chains is

(43) $$\sum_{s}\frac{3{\left(r\left(s\right)-X\left(s\right)\right)}^{2}}{2b^{2}N_{e}^{2}}$$

Here $r_{\alpha }\left(s\right)=x_{\alpha }\left(s\right)/\lambda_{\alpha }$ and $X_{\alpha }\left(s\right)$ (α=x,y,z) are undeformed coordinates of two ends of the *s*-th virtual chain. As shown in [3], with this choice of the potential of virtual chains, they do not contribute to the elastic stress of the polymer network, which is determined only by the contribution of all real polymer chains.

### 3.1. Physics of Entangled Network Deformation

Consider the case of a highly entangled network, $N/N_{e}\gg 1$, with many entanglements per network chain. As shown in [28], the network deforms affinely like a solid only at spatial scales exceeding the affine length $R_{\alpha }^{aff}$, see Figure 3. On shorter scales, chains take on liquid-like conformations. In the case of anisotropic deformation of the gel by the ratios $\lambda_{\alpha }$ along the axes α=x,y,z, the affine length is also anisotropic and depends on the direction α:

(44) $$R_{\alpha }^{aff}≃\lambda_{\alpha }a_{0}$$

We define an affine strand of the size $R_{\alpha }^{aff}$ directed along the axis α, which consists of $N_{\alpha }^{aff}$ monomers and deforms by the stretching ratio $\lambda_{\alpha }$. The number of chain entanglements does not change upon network deformation; therefore, the diameter of the deformed tube $a_{\alpha }$ is equal to the size of the entangled segment of $N_{e}$ monomers. In the stretched network this segment is a section of a longer stretched affine strand of size $R_{\alpha }^{aff}>a_{\alpha }$:

(45) $$a_{\alpha }≃R_{\alpha }^{aff}\left(N_{e}/N_{\alpha }^{aff}\right)$$

Fluctuations of the affine strand $b{\left(N_{\alpha }^{aff}\right)}^{1/2}$ are limited by entanglements and therefore equal to the tube diameter

(46) $$b{\left(N_{\alpha }^{aff}\right)}^{1/2}≃a_{\alpha }$$

The solution of Equations (45) and (46) is

(47) $$N_{\alpha }^{aff}=\lambda_{\alpha }N_{e}$$

Thus, the entanglement efficiency decreases with a network stretching, and the entanglements are not effective at $N_{\alpha }^{aff}>N$; such an entangled network behaves like a phantom one. The free energy of the entangled network is estimated as the elastic energy of all its stretched entangled segments,

(48) $$\frac{F_{e}}{V}≃\frac{\rho kT}{N_{e}}\sum_{\alpha }\frac{{\left(R_{\alpha }^{aff}\right)}^{2}}{a_{\alpha }^{2}}≃\frac{\rho kT}{N_{e}}\sum_{\alpha }\lambda_{\alpha }$$

A more rigorous estimate for the free energy of the non-affine tube model with the number of monomers of the chain $N\gg N_{e}$ gives [29]

(49) $$\frac{F_{e}}{V}=\frac{\rho kT}{2N_{e}}\sum_{\alpha }\left(\lambda_{\alpha }+\frac{1}{\lambda_{\alpha }}\right),$$

This expression includes an additional to Equation (48) contribution ∼$1/\lambda_{\alpha }$ describing the compression of the chain in the tube.

### 3.2. Slip-Tube Model

This model generalizes the nonaffine tube model by taking into account the slippage of chains along the tube contour. In the case of anisotropic deformation of the network, its chains are more stretched in the direction of maximum extension relative to other directions. The increased chain tension in this direction draws the chain from the tube segments along these directions. The redistribution of the chain length between different sections of the tube is taken into account by renormalization of parameters of the non-affine tube model for each direction of deformation

(50) $$\lambda_{\alpha }\to \lambda_{\alpha }/g_{\alpha }^{1/2},\;N\to N/g_{\alpha }$$

The parameters $g_{\alpha }$ are normalized by the condition of preserving the full length of the chain,

(51) $$\sum_{\alpha }g_{\alpha }=3$$

and in the case of uniaxial deformation, Equation (3), there is only one independent redistribution parameter $g_{z}$:

(52) $$g_{x}=\left(3-g_{z}\right)/2$$

In addition to the above renormalization of Equation (49), the free energy of this model has an entropic contribution taking into account the chain slippage along the tube,

(53) $$\frac{F_{e}}{V}=\frac{\rho kT}{2N_{e}}\sum_{\alpha }\left(\frac{\lambda_{\alpha }}{g_{\alpha }^{1/2}}+\frac{g_{\alpha }^{1/2}}{\lambda_{\alpha }}\right)-\frac{\rho kT}{3N_{e}}\sum_{\alpha }\ln g_{\alpha },$$

The parameters $g_{\alpha }$ are found by minimizing the free energy (62), and the minimization equation reads as

(54) $$h_{z}\left(g_{z}\right)=h_{x}\left(g_{x}\right),\;h_{\alpha }\left(g_{\alpha }\right)=\frac{\partial }{\partial g_{\alpha }}\frac{F_{e}}{\nu kT}$$

The elastic stress of the slip-tube model is

(55) $$\frac{\sigma_{\alpha \alpha }^{e}}{\nu kT}=\frac{\lambda_{\alpha }}{\nu kT}\frac{dF_{e}}{d\lambda_{\alpha }}.$$

The effect of entanglements substantially depends on the degree of network stretching, and the chains of strongly stretched networks become effectively phantom. Such a crossover from entangled to affine behavior is usually described using the assumption of additivity of the phantom and affine contributions to the free energy:

(56) $$F=F_{ph}+F_{e}$$

This assumption also leads to the additivity of the corresponding contributions to the elastic tensor,

(57) $$\sigma_{\alpha \alpha }=\sigma_{\alpha \alpha }^{ph}+\sigma_{\alpha \alpha }^{e}$$

In the case of uniaxial network deformation, Equation (3), a convenient representation of stress is the Mooney ratio of the total stress $\sigma_{zz}-\sigma_{xx}$ to the functional dependence $\lambda^{2}-\lambda^{-1}$ predicted by phantom network models:

(58) $$f^{*}\left(\lambda^{-1}\right)=\frac{\sigma_{zz}-\sigma_{xx}}{\lambda^{2}-\lambda^{-1}}$$

As was shown in Ref. [3], the Mooney plot of the experimental data is well described by the slip tube model.

### 3.3. Crossover from Unentangled to Entangled Regimes

The Mooney-Rivlin dependence (58) is usually fitted by the phenomenological equation

(59) $$f^{*}\left(\lambda^{-1}\right)≃2C_{1}+2C_{2}/\lambda$$

It is usually assumed that the parameter $2C_{1}$ is only due to chemical cross-links, while the parameter $2C_{2}$ includes all of the entanglement contributions. According to the non-affine tube model, part of the entanglement contribution is also included in the parameter $2C_{1}$ [6].

In this section, we study the concentration dependence of the Mooney-Rivlin coefficients. Although the free energy additivity approximation (56) can be used to describe the polymer network in the melt, a refined theory should be developed to find the Mooney dependence in the entire concentration range. The importance of such a study is emphasized by the fact that the networks prepared near the crossover from unentangled to entangled regimes are a fairly typical case.

Consider a network, consisting of ν Gaussian chains with *N* monomers whose endpoints are displaced affinely with the global deformation of the network. We assume that each chain of this network is confined in an effective “non-affinely deformed” tube, described by the virtual chain’s potential (43). The free energy of this model is calculated as the sum of contributions of all its strands and is found in Appendix C:

(60) $$\frac{F}{\nu kT}=\sum_{\alpha }\left(\frac{\lambda_{\alpha }^{2}-1}{2}\frac{k_{\alpha }}{\tanh k_{\alpha }}+\ln\frac{\sinh k_{\alpha }}{k_{\alpha }}\right),$$

where $k_{\alpha }=N/\left(N_{e}\lambda_{\alpha }\right)$. In the “phantom” limit $N\ll N_{\alpha }^{aff}=\lambda_{\alpha }N_{e}$ Equation (60) turns into the free energy of affine network model, Equation (4). In the limit of strongly entangled networks, $N\gg N_{\alpha }^{aff}$, this expression reproduces the free energy of the non-affine tube model, Equation (49). The first term in Equation (60) can be rewritten as

(61) $${\left(\frac{\lambda_{\alpha }R_{0}}{R_{fl,\alpha }}\right)}^{2}$$

where $R_{0}=bN^{1/2}$ is the chain size under network preparation conditions and $R_{fl,\alpha }$ is the fluctuation size in the α-direction, equal to $R_{0}$ in the phantom regime and the tube diameter $a_{\alpha }$ in the entangled regime. The logarithmic term in this expression describes the entropy of the chain compression in the tube.

By renormalizing the parameters of this model (50) to take into account the effect of chain redistribution in the entangled tube, and adding the contribution of entropy of slippage along the tube (53), we arrive at the expression for free energy

(62) $$\frac{F}{\nu kT}=\sum_{\alpha }\left(\frac{\lambda_{\alpha }^{2}-g_{\alpha }}{2}\frac{k_{\alpha }}{\tanh k_{\alpha }}+g_{\alpha }\ln\frac{\sinh k_{\alpha }}{k_{\alpha }}-\frac{N}{3N_{e}}\ln g_{\alpha }\right),$$

Solving equations (54) for this model, we find the stress (55) in the polymer network, which determines the Mooney-Rivlin dependence (58). The parameter $C_{2}\left(\phi \right)$ of this dependence is determined by the expression

(63) $$2C_{2}\left(\phi \right)=\frac{df^{*}\left(\lambda^{-1}\right)}{d\lambda^{-1}}.$$

**Figure 4:**
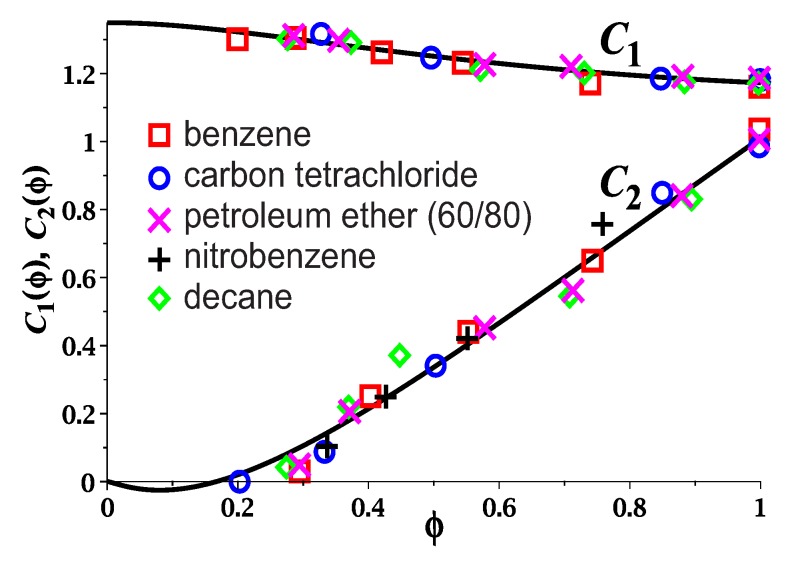
Dependence of the Mooney-Rivlin coefficients $C_{1}\left(\phi \right)$ (upper curve) and $C_{2}\left(\phi \right)$ (lower curve) on the volume fraction of polymer ϕ: theory (solid curves) and experimental data (points, Kg/cm${}^{2}$) for swollen rubber [30].

We plot the dependences $C_{1}\left(\phi \right)$ and $C_{2}\left(\phi \right)$ (63) for $\lambda^{-1}=0.7$ and $N/N_{e}=2.4$ in Figure 4. The parameter $C_{1}$ of the Mooney-Rivlin dependence (59) weakly depends on the polymer volume fraction ϕ. In accordance with the experimental data [30], the dependence of the parameter $C_{2}$ on ϕ is almost linear, and $C_{2}$ vanishes at ϕ≃0.2. The vanishing of $C_{2}$ at finite ϕ is related to the transition between phantom and entangled regimes. When the polymer swells, its chains move away from each other, which leads to a weakening of the effective topological potential that holds the chains in the tube. Chain fluctuations in this potential increase with the swelling and can reach the fluctuations of the phantom chain. This concentration corresponds to the disappearance of the contribution of entanglements to the network elasticity and the vanishing of the Mooney-Rivlin coefficient, $C_{2}=0$.

## 4. Heterogeneities in Polymer Networks

Network inhomogeneity is a common feature of polymer networks and gels due to the randomness of the crosslinking process and the presence of additional topological defects such as dangling chain ends, cross-linker shortcuts, and chains forming loops. The origin of nanostructural inhomogeneities and their characterization by light, neutron, and X-ray scattering as well as by NMR spectroscopy and optical, electron, and X-ray microscopies is reviewed in Ref. [31], and the main methods of their study are outlined in the review [32]. The first attempts to take such heterogeneities into account were made by using the model of randomly cross-linked networks containing fractal regions, such as percolation clusters [33]. Networks with fractal heterogeneities can be swollen and deformed by unfolding the fractal regions without significant elastic entropy penalty [34]. It is shown that strong heterogeneities lower the shear modulus of the network if a part of strands is so short to be considered rigid and not able to deform. Networks with a large number of such very short strands have higher breaking energies.

The effect of entanglements on heterogeneities in polymer networks is studied in Ref. [35] using molecular dynamics simulations of polymer networks made by either end-linking or randomly crosslinking a melt of linear precursor chains. The end-linking leads to nearly ideal monodisperse networks, while random cross-linking produces strongly polydisperse networks. It is shown that the microscopic strain response, the diameter of the entanglement tube, and stress–strain relation weakly depend on the linking process by which the networks were made.

The replica method was used in Ref. [28] to calculate the characteristic size and amplitude of the spatial nonuniformities of the network due to defects of its structure and topological restrictions. Using this method it is shown that inhomogeneities can arise as consequences of a stretching of polymer networks [36]. Although the replica theory [8] provides a complete solution of the statistical mechanics of polymer gels, it uses replica trick which is unfamiliar to the majority of people in the polymer community. A more intuitive phenomenological approach capturing all the main physical ingredients of the complete theory is developed in Ref. [37].

### 4.1. Theory of Heterogeneities in Polymer Networks

In this section, we review the main results of the theory of random spatial heterogeneities developed in swollen and deformed polymer networks [38]. The non-triviality of this problem stems from the fact that information about network structure is “encrypted” in the pattern of cross-links joining polymer chains, which represent a very small fraction of the network volume. The initial crosslinking pattern is reproduced only partially due to thermal fluctuations arising in the new equilibrium state after crosslinking. Conformations of polymer strands in such networks with fixed topological structure can be varied in a wide range depending on experimantal conditions.

The density profile of monomers in the polymer network can be recovered from the Fourier component of the deviation of the density from its average value ρ:

(64) $$\rho \left(x\right)=\rho +\int \tilde{\rho }\left(q\right)e^{iqx}\frac{dq}{{\left(2\pi \right)}^{3}}=\rho_{eq}\left(x\right)+\delta \rho \left(x\right)$$

Here, δρ(x) are random deviations (due to thermal fluctuations) of the density from the equilibrium density profile $\rho_{eq}\left(x\right)$ describing spatial inhomogeneities in polymer networks. The fluctuation contribution to the free energy of the network with a given distribution of monomer density can be represented as the sum of the entropy contribution and the (osmotic) contribution of volume interactions ∼v:

(65) $$F\left[\tilde{\rho }\right]=\frac{kT}{2}\int \left[\frac{{\left|\tilde{\rho }\left(q\right)-\tilde{n}\left(q\right)\right|}^{2}}{\tilde{g}\left(q\right)}+v{\left|\tilde{\rho }\left(q\right)\right|}^{2}\right]\frac{dq}{{\left(2\pi \right)}^{3}}$$

Here $\tilde{n}\left(q\right)$ is the density profile in the “elastic reference state” [37], maximizing the entropy of the polymer network. The density $\tilde{n}\left(q\right)$ is determined by the molecular structure of the network and it vanishes in the short-wavelength limit $q\gg R^{-1}$, since solid-state degrees of freedom are determined only at length scales exceeding the monomer fluctuation radius *R*. On a smaller scale, the gel has liquid degrees of freedom, contributing to the temperature structural factor

(66) $$\tilde{g}\left(q\right)=\rho N\left[\frac{1}{Q^{2}/2+{\left(4Q^{2}\right)}^{-1}+1}+\frac{2Q^{2}}{{\left(1+Q^{2}\right)}^{2}{\left(\lambda \cdot Q\right)}^{2}}\right]$$

The first term in square brackets determines the contribution of the liquid degrees of freedom of the polymer network. The dimensionless wavevector Q=Rq is normalized by the monomer fluctuating radius, and the vector λ·Q has components $\left(\lambda_{x}Q_{x},\lambda_{y}Q_{y},\lambda_{z}Q_{z}\right)$. At large Q≫1 the term $Q^{2}/2$ in the denominator of the first term of the right hand side of Equation (66) gives the usual Lifshitz entropy of polymer solutions, $\tilde{g}\left(q\right)=2\rho N/Q^{2}$ [39]. The term ${\left(4Q^{2}\right)}^{-1}$, first introduced by de Gennes for heteropolymer networks [40], describes the suppression of density fluctuations on length scales larger than the monomer fluctuation radius *R*. The second term in square brackets in Equation (66) determines the contribution of solid-state degrees of freedom of the polymer network. It remains finite in the long-wavelength limit and retains its angular dependence on the anisotropic deformation even in the limit q→0.

Equilibrium monomer density profile is found by minimizing the free energy (65)

(67) $${\tilde{\rho }}_{eq}\left(q\right)=\frac{\tilde{n}\left(q\right)}{1+v\tilde{g}\left(q\right)}$$

As can be seen from Equation (67), in good solvent with positive excluded volume v>0, the equilibrium density profile is more homogeneous than that of the corresponding elastic reference state. The excluded volume interaction suppresses not only thermal fluctuations in the polymer network, but also frozen-in spatial inhomogeneities. Although the static spatial fluctuations are permanent inhomogeneities, they can reversibly increase and diverge at the spinodal line, at which $v\tilde{g}\left(0\right)=-1$. This observation is experimentally confirmed in Ref. [41].

In (ergodic) heterogeneous systems, there are two types of averages, i.e., the *thermal* or *time averages* and *ensemble* or *space averages*, denoted by $\langle X\rangle$ and $\bar{X}$, respectively. The Fourier component of the thermal correlator of density fluctuations $\delta \rho \left(x\right)=\rho \left(x\right)-\rho_{eq}\left(x\right)$ is found by averaging with the Gibbs weight $e^{-F\left[\tilde{\rho }\right]/kT}$, Equation (65):

(68) $$\tilde{D}\left(q\right)=\langle {\left|\delta \tilde{\rho }\left(q\right)\right|}^{2}\rangle =\frac{\tilde{g}\left(q\right)}{1+v\tilde{g}\left(q\right)}$$

Therefore, in addition to permanent spatial heterogeneities, a polymer network undergoes thermal dynamical density fluctuations which diverge at the spinodal line.

All information about the heterogeneity of the molecular structure of the network is contained in the monomer density $\tilde{n}\left(q\right)$ in the “elastic reference state”, which can be considered as a random variable characterized by a correlator

(69) $$\nu \left(q\right)=\bar{\tilde{n}\left(q\right)\tilde{n}\left(-q\right)}=\frac{1}{{\left(1+Q^{2}\right)}^{2}}\left[6\rho N+\frac{9}{\lambda_{x}\lambda_{y}\lambda_{z}}{\tilde{S}}^{\left(0\right)}\left(\lambda \cdot q\right)\right]$$

Therefore, it is important to understand the physical meaning of the different terms of this expression:

Each of two factors ${\left(1+Q^{2}\right)}^{-1}$ describes the thermal “smearing” of the density response to random stresses in the polymer network. The first term in square brackets comes from the correlator of frozen random stresses, which exist even in a homogeneous polymer network. Such stresses appear due to heterogeneities in the distribution of cross-links. The second term in brackets in Equation (69), which is proportional to the correlator of the affine deformed density pattern in the reference state $S^{\left(0\right)}\left(\lambda \cdot q\right)$, strongly depends on network preparation conditions.

(1) In the case of cross-linking in a melt, density fluctuations are suppressed and ${\tilde{S}}^{\left(0\right)}\left(q\right)=0$. The fact that $\nu \left(q\right)$ does not vanish upon swelling of such a network, despite the absence of density fluctuations at the preparation conditions, means that there are still inhomogeneities in the crosslink density, which manifest themselves upon swelling.

(2) In the case of instant cross-linking of a semi-dilute polymer solution, ${\tilde{S}}^{\left(0\right)}\left(q\right)$ is given by the Ornstein-Zernicke expression

(70) $${\tilde{S}}^{\left(0\right)}\left(q\right)=\langle {\left|\delta {\tilde{\rho }}^{\left(0\right)}\left(q\right)\right|}^{2}\rangle =\frac{\rho^{\left(0\right)}N}{v^{\left(0\right)}\rho^{\left(0\right)}N+Q^{2}/2},$$

which is finite for any second virial coefficient $v_{0}$ at network preparation conditions.

(3) In case of equilibtium chemical cross-linking, the total structure factor of the gel in the state of preparation is determined by the expression for the polymer liquid, Equation (70), with renormalized excluded volume parameter, $v^{\left(0\right)}\to v_{ren}^{\left(0\right)}=v^{\left(0\right)}-{\left(\rho^{\left(0\right)}N\right)}^{-1}$. The decrease in $v^{\left(0\right)}$ is due to the fact that the monomers forming cross-links give an additional attractive contribution to the effective second virial coefficient $v_{ren}^{\left(0\right)}$. The amplitude of heterogeneities increases when approaching the *cross-link saturation threshold* at $v^{\left(0\right)}\rho^{\left(0\right)}N=1$ at which both the structure factor $S^{\left(0\right)}\left(q\to 0\right)$ and the characteristic size of heterogeneities at preparation conditions

$$\xi ≅R/\sqrt{v^{\left(0\right)}\rho^{\left(0\right)}N-1}$$

diverge.

The theory also predicts the appearance of a maximum degree of spatial gel inhomogeneity at a critical polymer network concentration, as confirmed by experiments on PAAm gels [42,43].

### 4.2. Scattering Intensity

The scattering intensity on wavevector q is proportional to the structure factor, which is given by the sum

(71) $$\tilde{S}\left(q\right)=\bar{\langle {\left|\tilde{\rho }\left(q\right)\right|}^{2}\rangle }=\tilde{D}\left(q\right)+\tilde{C}\left(q\right)$$

of contributions of the thermal fluctuations, Equation (68), and the inhomogeneous equilibrium density variations due to defects of the topological structure of the network,

(72) $$\tilde{C}\left(q\right)=\bar{{\left|{\tilde{\rho }}_{eq}\left(q\right)\right|}^{2}}=\frac{\nu \left(q\right)}{{\left[1+v\tilde{g}\left(q\right)\right]}^{2}}$$

where bar means ensemble average.

The experimental data can be visualized using contour plots of neutron scattering from random inhomogeneities of network structure in anisotropic-deformed swollen gels [44]. Under uniaxial gel stretching, an increase in the scattered intensity on small wave vectors *q* in the stretching direction is observed, which is enveloped by elliptical patterns at larger *q* values with a maximum oriented normal to this axis. This behavior is opposite of what is expected in theories assuming only thermal fluctuations and is called “abnormal butterfly patterns”. The elliptical patterns at large wavevector *q* originate from the correlator of static inhomogeneities, Equations (72) and (69), which contains the term $S_{0}\left(\lambda \cdot q\right)$ that “remembers” the affinely deformed inhomogeneous structure of the network. The butterfly patterns along the stretching axis in the small *q* range is due to strong angular dependence of the thermal structure factor, (68). This function comes into numerator of the thermal correlator in Equation (68), describing “normal butterfly patterns”. At high cross-link concentrations the correlator of static inhomogeneities gives the main contribution to the scattering intensity. Since the function $\tilde{g}\left(q\right)$ is included in the denominator of Equation (72), this expression describes the so named “abnormal butterfly patterns”. As a result, under uniaxial extension, a crossover from normal to abnormal butterfly scattering patterns occurs with an increase of the strength of inhomogeneity or the swelling ratio [45]. In addition to such butterfly scattering patterns, the theory also predicted “Lozenge” patterns if only part of all network chains, i.e., their deuterated fragments, could scatter neutrons, in accordance with scattering experiments [46].

The static inhomogeneity in poly (N-isopropyl acrylamide) gel (PNIPA) has been investigated in Ref. [47] by the methods of small-angle neutron scattering (SANS) and neutron spin echo. The obtained SANS scattering amplitude $\tilde{S}\left(q\right)$ was successfully decomposed into the thermal and static components, respectively, $\tilde{D}\left(q\right)$ and $\tilde{C}\left(q\right)$ in Equation (71). It was revealed that $\tilde{C}\left(q\right)$ becomes dominant in the *q*-region, where the abnormal butterfly scattering under stretching is observed. As the temperature increases toward the temperature for volume phase transition, $\tilde{C}\left(q\right)$ approximated by the square of the Lorentzian shape increases more drastically than $\tilde{D}\left(q\right)$ of the Lorentzian shape. These experimental findings are also well described in the theoretical framework of this section.

In Ref. [48] gels prepared by two cross-linking methods were studied using SANS technique. One is chemical cross-linking with BIS and the other is gamma-ray cross-linking of a PNIPA solution. It is shown that the degree of the inhomogeneity is much larger in chemically cross-linked gels than in the gamma-ray cross-linked gels. The experimental data are in quantitative agreement with predictions of the theory of scattering intensity $\tilde{S}\left(q\right)$ on chemically and instantaneously cross-linked gels, respectively.

### 4.3. Heterogeneities in Charged Gels

A study of the structure factor of weakly charged polyelectrolyte gels under uniaxial stretching was performed by Mendes et al. [49], who observed after introducing ions to the gel, the disappearance of the “butterfly pattern” in the small angle scattering intensity, as well as an increase in the scattering intensity in the direction perpendicular to the gel stretching. The origin of this maximum has been elucidated in SANS experiment by Shibayama et al. who studied the deformed state of weakly charged polymer gels PNIPA/AAc [50]. In this experiment, an anisotropic scattering maximum is observed, which indicates that the spatial distribution of the charged groups changes as a result of gel deformation and therefore is strongly coupled with the static inhomogeneities.

All the observed patterns of SANS intensity were well reproduced using a generalization of the above theory to the case of charged polymer networks [51]. The only effect of electrostatic interactions is to replace the excluded volume parameters $v^{\left(0\right)}$ and *v* with effective virial coefficients for both the final state and for the state of preparation:

$$\begin{array}{cc} v\; & \to v\left(q\right)\;\equiv v+1/s_{DH}\left(q\right),\\ v^{\left(0\right)} & \to v^{\left(0\right)}\left(q\right)\equiv v^{\left(0\right)}+1/s_{DH}^{\left(0\right)}\left(q\right) \end{array}$$

where

$$\frac{1}{s_{DH}\left(q\right)}=\frac{4\pi l_{B}\alpha^{2}}{\kappa^{2}+q^{2}}$$

is the inverse of the Debye-Hückel structure factor, $\kappa^{-1}$ is the Debye screening length, α is the degree of ionization (fraction of charged monomers) and $l_{B}$ is the Bjerrum length. $s_{DH}^{\left(0\right)}\left(q\right)$ is obtained by substituting $\kappa =\kappa^{\left(0\right)}$ and $\alpha =\alpha^{\left(0\right)}$ into the above equation. As shown in Ref. [52], the above theoretical prediction for $S\left(q\right)$ well reproduces the observed scattering intensity functions of wekly charged PNIPA/AAc gels.

In general, an introduction of cross-links to a polymer solution leads to an increase in the scattering intensity due to static inhomogeneities. However, a reverse phenomenon, called the “inflection” in scattering intensity, was predicted by the above theory [53] and observed in weakly charged gels and polymer solutions [54]. While the gel becomes more inhomogeneous with increasing the degree of cross-linking in a good solvent, the inhomogeneities can be suppressed in a poor solvent, although in a relatively small regions of cross-link concentration. However, this phenomenon is interesting due to its physical significance.

### 4.4. Good Solvent: Scaling Approach

Earlier in this paper, we considered the networks with Gaussian chains obtained in the melt or the θ-solution of linear chains. In general, polymer networks can also be obtained by crosslinking semi-diluted polymer solutions. The networks can be placed in a good solvent, in which the polymer chains swell compared to the case of theta solvent. This section explains the basic ideas of the scaling approach to describing networks prepared/swollen in a good solvent.

The mean-field approach can be adapted to descripbe the elasticity of such gels [55] using the well-known de Gennes blob picture of semi-dilute solutions [56]. The key idea is the spatial scale separation: while static density inhomogeneities exist only on scales comparable to or larger than the monomer fluctuation radius *R*, thermal density fluctuations are dominated by smaller scales and are quite similar to those in semi-dilute polymer solutions.

Consider a gel prepared by random cross-linking of chains in a semi-dilute polymer solution in a good solvent at the monomer density $\rho^{\left(0\right)}$ that is swollen to density $\rho <\rho^{\left(0\right)}$. The monomer density fluctuations should be taken into account both in the initial (where the gel was prepared) and in the final (where it is being studied) states of the gel. On length scales shorter than the correlation lengths in these states,

(73) $$\xi^{\left(0\right)}=b^{-5/4}{\left(\rho^{\left(0\right)}\right)}^{-3/4}\;\;\;\;\;\;\;\;\mathrm{and}\;\;\;\;\;\;\;\;\xi =b^{-5/4}\rho^{-3/4},$$

density fluctuations are large, and the gel behaves like a polymer solution (“liquid-like” regime). On scales exceeding the blob sizes (the corresponding wave vectors $q^{\left(0\right)}=1/\xi^{\left(0\right)}$ and q=1/ξ), density fluctuations are small, and the mean-field description with appropriately renormalized parameters can be used [8]

$$\begin{array}{cc} b^{\left(0\right)} & \to b_{ren}^{\left(0\right)}=b{\left(\rho^{\left(0\right)}b^{3}\right)}^{-1/8},\\ b\; & \to b_{ren}=b{\left(\rho b^{3}\right)}^{-1/8},\;\;\;\;\lambda_{\alpha }\to \lambda_{\alpha }b_{ren}^{\left(0\right)}/b_{ren} \end{array}$$

where the subscript ${}_{ren}$ stands for renormalized values that differ from the “bare” ones. The renormalized second virial coefficients $v_{ren}^{\left(0\right)}$ and $v_{ren}$ are:

(74) $$v^{\left(0\right)}\to v_{ren}^{\left(0\right)}=b^{3}{\left(\rho^{\left(0\right)}b^{3}\right)}^{1/4},\;\;v\to v_{ren}=b^{3}{\left(\rho b^{3}\right)}^{1/4}$$

Equations (73) and (74) complete the renormalization of the mean-field theory: in order to describe a gel in a good solvent on length scales larger than the thermal correlation length, one only have to replace the bare parameters in the previously derived expressions for the free energy, correlation functions, etc., by their renormalized values.

### 4.5. Amplification of Cross-Linking Density Pattern

Is it possible to “write” some “useful” information in a polymer network cross-link pattern, and under what conditions it can be “read” back? The answer to this question is given in Ref. [57]. After the formation of a homogeneous (on length scales large compared to its “mesh” size *R*) network, large-scale patterns can be generated in it by further cross-linking followed by swelling (and possibly stretching) of the network, which leads to a nonuniformly swollen gel. This additional cross-linking can be done by adding light-sensitive cross-links to the transparent network. By focusing the laser beam in the regions inside the gel, it is possible to “write” information into the gel structure in the form of 2D or 3D patterns of cross-link density.

It was shown that although such information is hidden at the preparation conditions, it can be reversibly “developed” by gel swelling, since unobservable variations of the cross-link density in the melt are transformed into observable variations of monomer density in the swollen gel. The gel regions with increased cross-link concentration can be considered as inclusions with enhanced elastic modulus. It has been shown that in swollen gels that stretch isotropically upon absorption of the solvent, the observed monomer density pattern is an affinity stretched initial cross-linking pattern. Such gels can serve as a magnifying glass that enlarges the initially written pattern without distorting its shape. The corresponding magnification factor can be very large in the case of super-elastic networks.

A strong enhancement of the contrast between the high and the low monomer density regions can be obtained by placing the gel in a poor solvent with a negative second virial coefficient v<0. The observable image becomes distorted, especially near the edges and corners of the pattern. Gel boundaries should be fixed in order to avoid its contraction. Gel contraction can also be prevented by focusing a laser beam only on a part of the localized pattern and heating it, resulting in a local change of the quality of solvent.

## 5. Discussion

Despite its more than half-century history, even now the theory of polymer network elasticity asks more questions than it gives answers. In this work, we tried to bridge the gap between the main developed approaches, which allowed us to give answers to some of these questions.

In real networks, there are both topological defects and typical loops, which are not sparsely distributed. Using the replica method, the cumulative effect of a large number of strongly overlapping typical loops of finite size can be described in the effective mean-field approximation. This approximation works for polymer networks due to large overlap parameter of network strands, $P^{\left(0\right)}\gg 1$, see Equation (12).

In the replica approach, the properties of polymer networks are described by the liquid-solid (sol-gel) order parameter $\varphi_{1}\left(\varsigma \right)$, which is actually not a parameter, but a function of the variable ς, and is determined by a complex integro-differential equation. The only, but very important exception is the elastic modulus, which is expressed through the value of the order parameter at ς=0, and the corresponding equations for $\varphi_{1}\left(0\right)$ become algebraic. This greatly simplifies the calculations of the elastic modulus of the network with an arbitrary number of cyclic fragments of finite size. In contrast to the classical theory of phantom networks, the mean field of loops, $\varphi_{1}\left(0\right)$ explicitly depends on the excluded volume parameter $v^{\left(0\right)}$ and the density of monomers $\rho^{\left(0\right)}$ at network preparation conditions. This dependence takes into account the limitations imposed by the packing of highly overlapping typical loops in real 3D space on the molecular structure of the network being formed.

The resulting elastic modulus of real networks has two main contributions, see Equation (37). The contribution of elastically effective network strands is taken into account in the classical model of phantom networks. The classical expression for this contribution is renormalized due to the presence of topological defects in the network—primary loops and cyclic fragments of finite size. The second contribution to the elastic modulus is always negative and gives an impact of a large number of typical loops of the network. It describes the effect of shunting of the elastically effective chains by finite size polymer loops and depends on the interaction of monomers at the preparation conditions of the network.

Both contributions to the network elastic modulus can be represented by the generalized combined chain model with an additional effective chain (see Figure 2b) that accounts the cumulative effect of finite size loops in real networks. The virtual chains transmit local stresses from the network fragments to the solid-state degrees of freedom of such soft solids. We established the connection of the distribution function of the lengths of virtual chains with the order parameter of the replica network model and calculated this distribution function for the exponential distribution of the lengths of the network strands.

We also proposed a generalization of the slip tube model of entangled polymer networks, which allows us to describe the crossover between the entangled and phantom regimes of network swelling. The dependence of the Mooney-Rivlin parameters $C_{1}$ and $C_{2}$ on the polymer concentration calculated for this model is in agreement with the experiments.

Although from the very beginning the heterogeneities were recognized as one of the most essential features of gels [58], it took long time to formulate their theoretical description due to complexity of this problem. Up to today, the understanding of the gel structure has greatly improved owing to both theoretical developments and a large number of experimental studies. Effect of cross-links on heterogeneous structure of polymer gels, abnormal butterfly patterns, microphase separation, and so on, are well understood with the aid of the theretical approaches discussed in Section 4.

The list of remaining questions is much longer:

Polymer networks can be obtained by crosslinking semidiluted polymer solutions. How does a strong nonlinear deformation of such networks occur in the phantom regime? How do such networks fracture when they are strongly stretched? The molecular theory (Lake-Thomas model) of the fracture of dry networks is constructed in Ref. [59]. What happens when compressing such networks? What is deformation mechanism of heterogeneous super-tough networks, kinetics of crack growth and phase transitions in such networks? Stress-induced microphase separation in multicomponent networks was studied in [60]. What is the effect of microphase separation on the elasticity of such networks?

These and many other questions are still awaiting answers.

## Appendix A. Replica Model of the Network

Consider a network prepared by random end-linking a solution of polydisperse linear chains by *f*-functional cross-links. Following the idea of $\varphi^{4}$ formulation of the excluded volume problem [56], we introduce n→0 component field, $\varphi \left(\hat{x}\right)$ in the replica space with components $\varphi_{i}$ (i=1,…,n), which is related to the monomer density in this space as

(A1) $$\rho \left(\hat{x}\right)=\frac{1}{2}\varphi^{2}\left(\hat{x}\right)=\frac{1}{2}\sum_{i=1}^{n}\varphi_{i}^{2}\left(\hat{x}\right).$$

Unlike the main text, in this Appendix the symbol *n* is used only to indicate the number of components of the field φ. The free energy $F_{m}$ of the network in the replica system is described by Hamiltonian

(A2) $$\begin{array}{cc} H\left[\varphi \right] & =\int \;\;d\hat{x}\left[\frac{1}{2}\mu \varphi^{2}+\frac{b^{2}}{2}{\left(\hat{\nabla }\varphi \right)}^{2}-\frac{z_{f}}{f!}\varphi_{1}^{f}\right]\\ \; & +\sum_{k}\int \;dx^{\left(k\right)}\frac{v^{\left(k\right)}}{8}{\left[\prod_{l\neq k}\int \;dx^{\left(l\right)}\varphi^{2}\right]}^{2}. \end{array}$$

Here, $\hat{\nabla }$ is the gradient of the variables $\hat{x}$, μ is monomer chemical potential and $z_{f}$ is the fugacity of *f*-functional cross-links. The last sum in Equation (A2) describes the two-body monomer interaction in the initial system characterized by the excluded volume parameter $v^{\left(0\right)}$ and in final systems (k=1,…,m) with the excluded volume parameters $v^{\left(k\right)}=v$.

In the mean-field approximation, the steepest descent value of the field $\varphi_{1}$ is found from the extremum of Hamiltonian in Equation (A13). The field $\varphi_{1}\left(\hat{x}\right)$ has the meaning of the order parameter of the liquid-solid (sol-gel) phase transition. The steepest descent solution $\varphi_{1}\left(\varsigma \right)>0$ depends on a single variable ς, Equation (6). At ς=0, the differential Equation (8) for this function becomes algebraic, an explicit solution of the minimum conditions is found

(A3) $$\varphi_{1}\left(0\right)={\left[\frac{\left(f-1\right)!}{\bar{N}z_{f}}\right]}^{1/\left(f-2\right)},$$

In the limit m→0, the dimensionless order parameter is determined by differential Equation (8). The free energy of the network depends only on $\varphi_{1}\left(0\right)$. Substituting Equation (A2) into Equation (5), we find the free energy of the network [8]:

(A4) $$F=V^{\left(0\right)}G\left[\sum_{\alpha }\frac{\lambda_{\alpha }^{2}}{2}+\ln\left(aN^{1/2}\right)\right]$$

with the elastic modulus

(A5) $$\frac{G}{kT}=\rho_{f}^{\left(0\right)}\left(\frac{f}{2}-1\right),$$

where $\rho_{f}^{\left(0\right)}$ is the cross-link concentration.

## Appendix B. Distribution of Virtual Chains

According to Equation (19) the distribution function $q\left(s\right)={\tilde{\chi }}^{\left(f-1\right)}\left(s\right)$ of the inverse number of monomers s=1/n can be presented as a convolution of f−1 distribution functions $\tilde{\chi }\left(s^{\prime }\right)$ of random variables $s^{\prime }=1/m$. The multiple convolution is defined recurrently as

(A6) $${\tilde{\chi }}^{\left(k\right)}\left(s\right)=\left({\tilde{\chi }}^{\left(k-1\right)}*\tilde{\chi }\right)\left(s\right)\equiv \int_{0}^{s}{\tilde{\chi }}^{\left(k-1\right)}\left(s-s^{\prime }\right)\tilde{\chi }\left(s^{\prime }\right)ds^{\prime }$$

The distribution function $\tilde{\chi }\left(s^{\prime }\right)$ of an individual branch of the tree is related to the distribution function $\pi \left(m\right)$ of the inverse values $m=1/s^{\prime }$ by an equation similar to Equation (25):

(A7) $$\tilde{\chi }\left(s^{\prime }\right)=\frac{1}{{\left(s^{\prime }\right)}^{2}}\pi \left(\frac{1}{s^{\prime }}\right)$$

Finally, the distribution function $\pi \left(m\right)$ of the monomer numbers m=N+n of one branch is a convolution of distribution functions of the monomer numbers of the network strands $P\left(N\right)$ and virtual chains $p\left(n\right)$,

(A8) $$\pi \left(m\right)=\left(P*p\right)\left(m\right)=\int_{0}^{m}P\left(m-n\right)p\left(n\right)dn$$

Equations (25) and (A6)–(A8) completely determine the distribution function $p\left(n\right)$ of the number of monomers *n* of virtual chains for a given distribution function $P\left(N\right)$ of the number of monomers *N* of real network strands. The only problem is solving this system of nonlinear integral equations. Here the replica method comes to the rescue, see Appendix A.

## Appendix C. Free Energy of Entangled Networks

The network free energy for given positions of attachment points X(s) of virtual chains to the affinely deformed background is

(A9) $$\begin{array}{cc} F\left[X\right] & =-\nu kT\sum_{\alpha }\ln\;\;\int Dx_{\alpha }\left(s\right)e^{-H_{\alpha }\left[x_{\alpha }\right]/kT}, \end{array}$$

(A10) $$\begin{array}{cc} \frac{H_{\alpha }\left[x_{\alpha }\right]}{kT} & =\int_{0}^{N}ds\left\{\frac{3{\dot{x}}_{\alpha }^{2}}{2b^{2}}+\frac{3{\left[x_{\alpha }\left(s\right)/\lambda_{\alpha }-X_{\alpha }\left(s\right)\right]}^{2}}{2b^{2}N_{e}^{2}}\right\}. \end{array}$$

The simplest way to calculate the above integral is to use the normal mode expansion of the chain trajectories, which satisfy the boundary conditions $x_{\alpha }\left(N\right)=\lambda_{\alpha }X_{\alpha }\left(N\right)$ and $x_{\alpha }\left(0\right)=\lambda_{\alpha }X_{\alpha }\left(0\right)$ ($X\left(s\right)$ are the positions of attachment points of virtual chains in the undeformed network):

(A11) $$x_{\alpha }\left(s\right)=\lambda_{\alpha }R_{\alpha }\frac{s}{N}+\sum_{\omega }{\tilde{x}}_{\alpha }\left(\omega \right)e^{i\omega s},\;\omega =\frac{2\pi k}{N},\;k=\pm 1,\cdots$$

where $R_{\alpha }=X_{\alpha }\left(N\right)-X_{\alpha }\left(0\right)$ and

(A12) $$X_{\alpha }\left(s\right)=\lambda_{\alpha }R_{\alpha }\frac{s}{N}+\sum_{\omega }{\tilde{X}}_{\alpha }\left(\omega \right)e^{i\omega s},\;{\tilde{x}}_{\alpha }\left(-\omega \right)={\tilde{x}}_{\alpha }^{*}\left(\omega \right),\;{\tilde{X}}_{\alpha }\left(-\omega \right)={\tilde{X}}_{\alpha }^{*}\left(\omega \right).$$

The mode with ω=0 is excluded from the summation because it corresponds to the translational displacement of the chain, which is prohibited in the network. Substituting the above expressions into Equation (A10) we get

(A13) $$\frac{H_{\alpha }\left[x_{\alpha }\right]}{kT}=\frac{3R_{\alpha }^{2}}{2b^{2}N}\lambda_{\alpha }^{2}+\frac{3}{2b^{2}}\sum_{\omega \neq 0}\left[\omega^{2}{\left|{\tilde{x}}_{\alpha }\left(\omega \right)\right|}^{2}+\frac{|{\tilde{x}}_{\alpha }\left(\omega \right)-\lambda_{\alpha }{\tilde{X}}_{\alpha }\left(\omega \right){|}^{2}}{N_{e}^{2}\lambda_{\alpha }^{2}}\right]$$

Changing the integration variables, ${\tilde{x}}_{i}\left(\omega \right)=\tilde{u}\left(\omega \right)+i\tilde{v}\left(\omega \right)$, we find

(A14) $$\begin{array}{cc} \frac{F\left[X\right]}{\nu kT} & =\sum_{\alpha }\left[\frac{3R_{\alpha }^{2}}{2b^{2}N}\lambda_{\alpha }^{2}+\frac{3\lambda_{\alpha }^{2}}{2b^{2}}\sum_{\omega >0}\frac{\omega^{2}{\left|{\tilde{X}}_{\alpha }\left(\omega \right)\right|}^{2}}{1+\omega^{2}N_{e}^{2}\lambda_{\alpha }^{2}}\right.\\ \; & \left.-\sum_{\omega >0}\ln\frac{2b^{2}N_{e}^{2}\lambda_{\alpha }^{2}}{3N(1+\omega^{2}N_{e}^{2}\lambda_{\alpha }^{2})}\right]. \end{array}$$

Here sums are taken only over positive ω since the only positive frequency components $\tilde{u}\left(\omega \right)$ and $\tilde{v}\left(\omega \right)$ can be taken as independent variables: the condition for the reality of the function x(s), Equation (A12), imposes restrictions $\tilde{u}\left(-\omega \right)=\tilde{u}\left(\omega \right)$ and $\tilde{v}\left(-\omega \right)=-\tilde{v}\left(\omega \right)$ on the real and imaginary parts of $\tilde{x}\left(\omega \right)$.

This free energy should be averaged over positions of attachment points, X(s), with correlation functions

(A15) $$\bar{{\tilde{X}}_{\alpha }\left(\omega \right){\tilde{X}}_{\beta }\left(-\omega \right)}=\frac{b^{2}}{3}\left(\frac{1}{\omega^{2}}+N_{e}^{2}\right)\delta_{\alpha \beta }$$

which corresponds to randomly placed brash of chain with two virtual chains of $N_{e}^{2}$ monomers

(A16) $$\bar{{\left[X\left(s\right)-X\left(s^{\prime }\right)\right]}^{2}}=b^{2}\left|s-s^{\prime }\right|+2N_{e}^{2}$$

Averaging the free energy (A14) with the correlation function, (A15) and changing the integration variables $\tilde{X}\left(\omega \right)\to \left(R,\tilde{p}\left(\omega \right),\tilde{q}\left(\omega \right)\right)$ when integrating over $X\left(\omega \right)$ we find

(A17) $$\begin{array}{cc} \frac{F}{\nu kT} & =\sum_{\alpha }\left[\frac{\lambda_{\alpha }^{2}}{2}+\sum_{\omega >0}\frac{\lambda_{\alpha }^{2}-1}{1+\omega^{2}N_{e}^{2}\lambda_{\alpha }^{2}}\right.\\ \; & \left.+\sum_{\omega >0}\ln\left(1+\frac{1}{N_{e}^{2}\lambda_{\alpha }^{2}\omega^{2}}\right)\right]+Const. \end{array}$$

Here all the terms which do not depend on the extension coefficients $\lambda_{\alpha }$ are absorbed in Const. The sum and the product can be expressed in terms of hyperbolic functions, see Equation (60).

## References

[1] Urayama K., Kohjiya S. (1998). Extensive stretch of polysiloxane network chains with random- and super-coiled conformations. Eur. Phys. J. B.

[2] Flory P.J. (1953). Principles of Polymer Chemistry.

[3] Rubinstein M., Panyukov S. (2002). Elasticity of Polymer Networks. Macromolecules.

[4] Panyukov S. (2019). Loops in Polymer Networks. Macromolecules.

[5] Ge T., Panyukov S., Rubinstein M. (2016). Self-Similar Conformations and Dynamics in Entangled Melts and Solutions of Nonconcatenated Ring Polymers. Macromolecules.

[6] Rubinstein M., Panyukov S. (1997). Nonaffine Deformation and Elasticity of Polymer Networks. Macromolecules.

[7] Deam R.T., Edwards S.F. (1976). The Theory of Rubber Elasticity. Philos. Trans. R. Sot. Lond. Ser. A.

[8] Panyukov S., Rabin Y. (1996). Statistical physics of polymer gels. Phys. Rep..

[9] Panyukov S.V., Rabin Y., Grosberg A.Y. (1989). Replica Field Theory Methods in Physics of Polymer Networks. Theoretical and Mathematical Models in Polymer Research.

[10] Panyukov S., Rabin Y., Fiegel A. (1994). Solid Elasticity and Liquid-Like Behaviour in Randomly Crosslinked Polymer Networks. Europhys. Lett..

[11] Edwards S.F. (1988). A field theory formulation of polymer networks. J. Phys..

[12] Cai L.-H., Panyukov S., Rubinstein M. (2015). Hopping Diffusion of Nanoparticles in Polymer Matrices. Macromolecules.

[13] Lang M., Kreitmeier S., Göritz D. (2007). Trapped Entanglements in Polymer Networks. Rubber Chem. Technol..

[14] Rubinstein M., Colby R.H. (2003). Polymer Physics.

[15] Schieber J.D., Horio K. (2010). Fluctuation in entanglement positions via elastic slip-links. J. Chem. Phys..

[16] Gusev A.A. (2019). Numerical Estimates of the Topological Effects in the Elasticity of Gaussian Polymer Networks and Their Exact Theoretical Description. Macromolecules.

[17] Helfand E., Tonelli A.E. (1974). Elastically Ineffective Polymer Chains in Rubbers. Macromolecules.

[18] Tonelli A.E., Laboratories B., Hill M. (1974). Elastically Ineffective Cross-Links in Rubbers. Macromolecules.

[19] Zhong M., Wang R., Kawamoto K., Olsen B.D., Johnson J.A. (2016). Quantifying the impact of molecular defects on polymer network elasticity. Science.

[20] Lin T.-S., Wang R., Johnson J.A., Olsen B.D. (2018). Topological Structure of Networks Formed from Symmetric Four-Arm Precursors. Macromolecules.

[21] Lin T.-S., Wang R., Johnson J.A., Olsen B.D. (2019). Revisiting the Elasticity Theory for Real Gaussian Phantom Networks. Macromolecules.

[22] Lang M. (2018). Elasticity of Phantom Model Networks with Cyclic Defects. ACS Macro Lett..

[23] Lange F., Schwenke K., Kurakazu M., Akagi Y., Chung U.I., Lang M., Sommer J.-U., Sakai T., Saalächter K. (2011). Connectivity and structural defects in model hydrogels: A combined proton NMR and Monte Carlo simulation study. Macromolecules.

[24] Zhou H., Woo J., Cok A.M., Wang M., Olsen B.D., Johnson J.A. (2012). Counting primary loops in polymer gels. PNAS.

[25] Wang R., Alexander-Katz A., Johnson J.A., Olsen B.D. (2016). Universal Cyclic Topology in Polymer Networks. Phys. Rev. Lett..

[26] Lang M., Miller T. (2020). Analysis of the Gel Point of Polymer Model Networks by Computer Simulations. Macromolecules.

[27] Panagiotou E., Kruger M., Millett K.C. (2013). Writhe and mutual entanglement combine to give the entanglement length. Phys. Rev. E.

[28] Panyukov S.V. (1989). Topology fluctuations in polymer networks. Sov. Phys. JETP.

[29] Panyukov S.V. (1988). Topological interactions in the statistical theory of polymers. Sov. Phys. JETP.

[30] Gumbrell S.M., Mullins L., Rivlin R.S. (1955). Departures of the elastic behaviour of rubbers in simple extension from the kinetic theory. Rubber Chem. Technol..

[31] Di Lorenzo F., Seiffert S. (2015). Nanostructural heterogeneity in polymer networks and gels. Polym. Chem..

[32] Seiffert S. (2016). Origin of Nanostructural Inhomogeneity in Polymer-Network Gels. Polym. Chem..

[33] Vilgis T.A., Heinrich G. (1992). The Essential Role of Network Topology in Rubber Elasticity. Angew. Makromol. Chem..

[34] Vilgis T.A., Sommer J.-U., Heinrich G. (1995). Swelling and fractal heterogeneities in networks. Macromol. Symp..

[35] Svaneborg C., Grest G.S., Everaers R. (2005). Disorder effects on the strain response of model polymer networks. Polymer.

[36] Panyukov S.V. (1993). Inhomogeneities as consequences of a stretching of polymer networks. JETP Lett..

[37] Panyukov S., Rabin Y. (1996). Polymer Gels: Frozen Inhomogeneities and Density Fluctuations. Macromolecules.

[38] Panyukov S.V. (2016). Theory of heterogeneities in polymer networks. Polym. Sci. Ser. A.

[39] Lifshitz I.M., Grosberg A.Y., Khokhlov A.R. (1978). Some problems of the statistical physics of polymer chains with volume interaction. Rev. Mod. Phys..

[40] de Gennes P.G. (1979). Effect of cross-links on a mixture of polymers. J. Phys. Lett..

[41] Matsuo E.S., Orkisz M., Sun S.-T., Li Y., Tanaka T. (1994). Origin of Structural Inhomogeneities in Polymer Gels. Macromolecules.

[42] Kizilay M.Y., Okay O. (2003). Effect of Initial Monomer Concentration on Spatial Inhomogeneity in Poly(acrylamide) Gels. Macromolecules.

[43] Kizilay M.Y., Okay O. (2004). Effect of swelling on spatial inhomogeneity in poly(acrylamide) gels formed at various monomer concentrations. Polymer.

[44] Bastide J., Leibler L., Prost J. (1990). Scattering by deformed swollen gels: butterfly isointensity patterns. Macromolecules.

[45] Onuki A. (1992). Scattering from deformed swollen gels with heterogeneities. J. Phys. II.

[46] Panyukov S.V. (1992). Microscopic theory of anisotropic scattering by deformed polymer networks. Sov. Phys. JETP.

[47] Koizumi S., Monkenbusch M., Richter D., Schwahn D., Farago B. (2004). Concentration fluctuations in polymer gel investigated by neutron scattering: Static inhomogeneity in swollen gel. J. Chem..

[48] Norisuye T., Masui N., Kida Y., Ikuta D., Kokufuta E., Ito K., Panyukov S., Shibayama M. (2002). Small angle neutron scattering studies on structural inhomogeneities in polymer gels: Irradiation cross-linked gels vs chemically cross-linked gels. Polymer.

[49] Mendes E., Schosseler F., Isel F., Boué F., Bastide J., Candau S.J. (1995). A SANS Study of Uniaxially Elongated Polyelectrolyte Gels. Europhys. Lett. (EPL).

[50] Shibayama M., Kawakubo K., Ikkai F., Imai M. (1998). Small-Angle Neutron Scattering Study on Charged Gels in Deformed State. Macromolecules.

[51] Rabin Y., Panyukov S. (1997). Scattering Profiles of Charged Gels: Frozen Inhomogeneities, Thermal Fluctuations, and Microphase Separation. Macromolecules.

[52] Shibayama M., Kawakubo K., Norisuye T. (1998). Comparison of the Experimental and Theoretical Structure Factors of Temperature Sensitive Polymer Gels. Macromolecules.

[53] Shibayama M., Ikkai F., Shiwa Y., Rabin Y. (1997). Effect of Degree of Cross-linking on Spatial Inhomogeneity in Charged Gels. I. Theoretical Predictions and Light Scattering Study. J. Chem. Phys..

[54] Ikkai F., Shibayama M., Han C.C. (1998). Effect of Degree of Cross-Linking on Spatial Inhomogeneity in Charged Gels. 2. Small-Angle Neutron Scattering Study. Macromolecules.

[55] Panyukov S.V. (1990). Scaling theory of high elasticity. Sov. Phys. JETP.

[56] de Gennes P.-G. (1979). Scaling Concepts in Polymer Physics.

[57] Panyukov S., Rabin Y. (2015). Cross-Linking Patterns and Their Images in Swollen and Deformed Gels. Macromolecules.

[58] Bastide J., Leibler L. (1988). Large-scale heterogeneities in randomly cross-linked networks. Macromolecules.

[59] Wang S., Panyukov S., Rubinstein M., Craig S.L. (2019). Quantitative Adjustment to the Molecular Energy Parameter in the Lake–Thomas Theory of Polymer Fracture Energy. Macromolecules.

[60] Panyukov S., Rubinstein M. (1996). Stress-Induced Ordering in Microphase-Separated Multicomponent Networks. Macromolecules.

